# Mast Cells in Peritoneal Fluid From Women With Endometriosis and Their Possible Role in Modulating Sperm Function

**DOI:** 10.3389/fphys.2019.01543

**Published:** 2020-01-09

**Authors:** Violetta Borelli, Monica Martinelli, Stefania Luppi, Francesca Vita, Federico Romano, Francesco Fanfani, Elisa Trevisan, Fulvio Celsi, Giuliano Zabucchi, Fabrizio Zanconati, Cristina Bottin, Giuseppe Ricci

**Affiliations:** ^1^Department of Life Sciences, University of Trieste, Trieste, Italy; ^2^Institute for Maternal and Child Health IRCCS “Burlo Garofolo”, Trieste, Italy; ^3^UOC di Ginecologia Oncologica, Dipartimento Scienze della Salute della Donna e del Bambino, Fondazione Policlinico Universitario A. Gemelli IRCCS, Rome, Italy; ^4^Department of Medicine, Surgery and Health Sciences, University of Trieste, Trieste, Italy

**Keywords:** endometriosis, infertility, mast cells, tryptase, sperm

## Abstract

Endometriosis is a local pelvic inflammatory process, frequently associated with infertility, with altered function of immune-related cells in the peritoneal environment. Mast cells are known to be key players of the immune system and have been recently involved in endometriosis and in infertility, with their mediators directly suppressing sperm motility. In this study, we evaluated the mast cell population and their mediators in the peritoneal fluid of infertile patients with endometriosis and their impact on human sperm motility. Peritoneal fluids, collected by laparoscopy from 11 infertile patients with endometriosis and 9 fertile controls were evaluated for the presence of mast cells, tryptase levels and their effect on sperm motility. Furthermore, an *in vitro* model of mast cells-sperm interaction in peritoneal fluid was set up, using LAD2 cell line as a mast cell model, and analyzed from a functional as well as a morphological point of view. Mast cell peritoneal fluid population and its main mediator, tryptase, is more represented in endometriosis confirming an involvement of these cells in this disease. Anyway it appears unlikely that tryptase enriched peritoneal fluid, which fails to inhibit sperm motility, could contribute to endometriosis associated infertility. Despite of this, sperm interaction with the mast cell surface (LAD2) induced a significantly mast cell-degranulation response in the peritoneal fluid from endometriosis which could directly modulate sperm function other than motility. This evidence lead us to suppose that there is, between these elements, an interrelationship which deserves further studies.

## Introduction

Endometriosis (EMS) is characterized by the presence and growth of endometrial tissue outside the uterus. It is a common disease among women of reproductive age. Population-based studies estimate a prevalence ranging between 1.8 and 3.3% in women aged 15–50 years (Morassutto et al., 2016). Endometriosis is frequently associated with infertility, even if affected women have an ovulatory activity or a mechanical patency of the fallopian tubes (Gupta et al., 2008; Máté et al., 2018).

The current consensus is that endometriosis is a local pelvic inflammatory process with altered function of immune-related cells in the peritoneal environment. There are recent studies which support this theory, suggesting that the peritoneal fluid of women with endometriosis contains an increased number of activated macrophages that secrete various local products, including growth factors, cytokines and possibly oxidation products (Capobianco and Rovere-Querini, 2013), implicating these factors in the development and progression of endometriosis and endometriosis-associated infertility. Although the contribution of specific immune cell subsets and their mediators to the onset and the course of the inflammatory process in endometrial lesions is still poorly understood, some evidence suggests that mast cells (MC) are crucially involved. Mast cells are known to be key players of the immune system, especially during allergic reactions. However, increasing evidence supports the involvement of MC also in the inflammatory process of EMS. High numbers of degranulated MC have been found in endometriotic lesions (Konno et al., 2003; Kempuraj et al., 2004; Sugamata et al., 2005; Anaf et al., 2006; Kirchhoff et al., 2012; Paula et al., 2015). Of note, this was not the case at unaffected sites of the peritoneum or eutopic endometrial tissue from EMS patients or healthy controls. Additionally, it has been shown that stem cell factor (SCF), the major growth differentiation and chemoattractant factor for MC, is found in higher concentrations in the peritoneal fluid (PF) of EMS patients (Osuga et al., 2000). Despite the possible MC role in the onset and/or the course of the pelvic inflammatory process of endometriosis, neither the amount of MC nor the level of their mediators has been evaluated in EMS-peritoneal fluid to the best of our knowledge.

Endometriosis has been also associated with infertility even in its early stages (Zondervan et al., 2018) before adhesion or anatomic distortion take place. The exact mechanism of endometriosis-associated infertility is not fully understood, although many possible causes have been suggested, among them the potential negative effects of cytokine-rich peritoneal fluid (flushing the tubal and endometrial environment) on sperm function (impairment of acrosome reaction and impairment of sperm motility) (Gupta et al., 2008; Broi et al., 2019). Since in humans there are no selective barriers separating the Fallopian tubes and the peritoneal cavity, PF can be present in the fertilization milieu (Harper, 1988). Although changes in PF volume, cell concentration, hormones, growth factors, cytokines and possibly free oxygen radicals production have been well characterized in endometriosis (Oral et al., 1996; Bedaiwy and Falcone, 2003; Bedaiwy et al., 2007; Nishida et al., 2011; Rižner, 2015), data about the effect of this fluid on sperm function are still controversial (Broi et al., 2019). Most of these studies suggest that substances found in the peritoneal fluid of patients with endometriosis could contribute to infertility through impairment of both sperm function and motion kinetics (Drudy et al., 1994; Zullo et al., 1994; Aeby et al., 1996; Oral et al., 1996; Liu et al., 2000; Luo et al., 2008; Xu et al., 2008; Mansour et al., 2009). However, other studies reported no adverse effect of PF in patients with endometriosis on sperm motility parameters, suggesting that peritoneal fluid in these patients cannot contribute to infertility (Chen et al., 1997; Sharma et al., 1999). Interestingly human recombinant tryptase, the main MC product in all human MC, including those found in the female genital tract (Yamanaka et al., 2000; Sivridis et al., 2001), has been shown to reduce human sperm motility *in vitro* (Weidinger et al., 2003), thus being a good candidate factor for influencing sperm fertilizing ability.

In this study, we evaluated for the first time the amount of MC and their main mediator (tryptase) in the peritoneal fluid of infertile patients with endometriosis and their impact on human sperm motility. Furthermore we evaluated mast cell-sperm interaction in an *in vitro* model by using a human mast cell line (LAD2).

## Materials and Methods

### Patients for PF Collection

Twenty women undergoing either diagnostic or operative laparoscopy at the Institute for Maternal and Child Health, IRCCS Burlo Garofolo, Trieste, Italy were enrolled in a case-control study. The study was reviewed and approved by the Ethical Committee of the Institute for Maternal and Child Health IRCCS Burlo Garofolo, Trieste, Italy (Prot. 1197/2015). Informed consent for participation in the study was obtained from all women.

The study group (EMS group) consisted in total of 11 infertile women, diagnosed with moderate/severe endometriosis (stage III-IV, *n* = 9) and minimal/mild endometriosis (stage I-II, *n* = 2) according to the revised criteria of the American Society for Reproductive Medicine ASRM (Canis et al., 1997). They had normal ovulation and no other identifiable female causes of infertility.

The control group (C group) consisted of a total of 9 fertile women, without endometriosis, subjected to laparoscopy to remove leiomyoma. Medical history and white blood cell count (WBC) were recorded for all the patients.

### PF Collection and Cytological Evaluation

Laparoscopy was performed, and all obtainable peritoneal fluid in the pouch of Douglas was aspirated (by a suction unit through a Teflon catheter) immediately after entering the abdominal cavity and collected in sterile plastic tubes. Blood-free samples were immediately transported to the laboratory where total cell numbers were counted (Coulter, Miami, FL, United States) and differential cell counts carried out using an optical microscope on Diff-Quik System (Medion Diagnostics, Gmbh, Düdingen, CH) and Toluidine Blue stained cytospin specimens (Cytospin 2, Shandon Inc., Pittsburgh, PA, United States) on by cytospin preparations after staining with Diff-Quick (Medion Diagnostics, Dudingen, Switzerland). Subsequently, 1 mL of PF was centrifuged (10 min, 600 *g*), filtered through a 0.22-μL membrane (Millipore, Bedford, MA, United States) and stored at −80°C until used (<12 months). The remaining volume of PF (at least 5 mL) was processed for cell blocks preparation by using 10% alcohol–formalin as a fixative agent as described by Shivakumarswamy et al. (2012). After paraffin embedding 3 μm thickness serial sections were prepared from this cell block and stained immunohistochemically for tryptase (see below).

One of the main advantages of the cell block technique is to obtain multiple sections for conventional stains and immunohistochemistry (Shivakumarswamy et al., 2012). Diagnostic sensitivity may be improved when cytology and cell block preparation are used in tandem.

### Immunocytochemical Staining for Tryptase

Immunohistochemistry was performed on formalin-fixed, paraffin-embedded cell block sections 3 μm thin using Ultravision Quanto Large Volume Detection System HRP Polymer (Bio Optica, Milan, Italy) pre-treated with Dewax and HIER Buffer H, pH 8. The sections were incubated for 30 min, at RT Temperature with anti Mast Cell Tryptase monoclonal antibody (15-MOB347 – Bio Optica Milan, Italy) diluted 1:600 in Thermo Scientific Antibody Diluent OP Quanto (Cat. # TA-125-ADQ). The primary antibody was omitted in negative controls (Figure 2).

Finally, the sections were incubated for 10 min with 3,3′ Diaminobenzidine chromogen (DAB – Dako Milan, Italy) and Mayer Hematoxylin to nuclear counterstain. Slides were scanned by D-Sight Microscope and Scanner (A. Menarini Diagnostic S.r.l. – Firenze, Italy), then analyzed by VISIA Imaging S.r.l. software.

### Protein Content

The PF protein content was quantitated with the Bradford method (Bradford, 1976) using bovine-serum-albumin (BSA) as standard and expressed in mg/mL.

### Tryptase Concentration and Enzymatic Activity

Tryptase, a tetrameric serine proteinase, is the major component of mast cell granules, and comprises up to 20% of the total protein of mast cells derived from lung, colon and skin tissue (Schwartz and Bradford, 1986; He and Xie, 2004). A colorimetric assay was used to determine the enzymatic activity of tryptase in the PF samples both from patients and controls (and LAD2 supernatants, see below) as previously described (Borelli et al., 2018). The assay is based on spectrophotometric detection of the chromophore p-nitroaniline (pNA) (Sigma-Aldrich, United States) after cleavage from the labeled substrate tosyl-gly-pro-lys-pNA. The free pNA was quantified using a microtiter plate reader at 405 nm (Biotek Instruments Inc). Samples were assayed in triplicate, and tryptase activity was expressed in (arbitrary unit of absorbance/mL) AU/mL.

Tryptase (Enzyme-Linked Immunosorbent Assay (ELISA) (USCN, Life Sciences Inc) immunoassay kit was used to determine the concentration of tryptase in PF samples and pools both from patients and controls. The assays were performed according to the manufacturer’s instructions and the results referred to a calibration curve expressed in ng/mL. Samples were assayed in triplicate.

### β-Hexosaminidase Enzyme Activity

β -hexosaminidase (β -hexo), another typical marker of mast cell granules, was assayed spectrophotometrically by the hydrolysis of 4-nitrophenyl N-acetyl-β-D-glucosaminide (Sigma-Aldrich, United States) as previously described (Gri et al., 2008; Medic et al., 2008). Samples were assayed in triplicate and β -hexo activity was expressed in (arbitrary unit of absorbance/mL) AU/mL.

### Semen Samples: Isolation of Motile Sperms

Fresh semen was collected (at the Assisted Reproduction Unit of the Institute for Maternal and Child Health IRCCS Burlo Garofolo and University of Trieste) from healthy subjects (*n* = 19, mean age + SD: 39.6 ± 7.3 years) who had given informed consent and had no history of diseases related to infertility. After complete liquefaction, the ejaculates were analyzed according to the standard semen parameters of the World Health Organization (WHO laboratory manual for the examination and processing of human semen, fifth edition. World Health Organization [WHO], 2010). Motility was determined by manual counting of at least 200 sperms observed under 400× phase contrast optics.

A leukocyte count was carried out by using standard peroxidase test, as described in the WHO laboratory manual. All subjects were asymptomatic for genitourinary infections. Only ejaculates with <1 × 10^6^ white blood cells/mL, total motility (World Health Organization [WHO], 2010: >40%) and progressive motility (World Health Organization [WHO], 2010: >32%) were used for the experiments.

For isolation of motile sperms, samples were processed using the swim-up technique to eliminate dead spermatozoa and other cells, including bacteria and leukocytes (Ricci et al., 2009). This methodology is based on the active movement of spermatozoa from the prewashed cell pellet into an overlaying medium. Ejaculates were washed (centrifuged at 500 *g* for 10 min) and resuspended in Human Tubular Fluid medium (HTFM, Irvine Scientific, United States), containing 0.05% BSA. The supernatant was discarded, the pellet was suspended in pre-warmed 0,5 mL of HTFM. The pellet was gently over-layered with HTFM in the tube, inclined at 45° and kept at 37°C for 45–60 min. A sterile Pasteur pipette was used to remove the supernatant containing > 95% actively motile sperms whose concentration ranged from 20 to 30 × 10^6^/mL.

### Effect of Peritoneal Fluid on Human Sperm Viability and Motility

Motility-enriched sperm samples (*N* = 19) were incubated in HTFM containing 0.05% BSA (as negative control) or peritoneal fluid in the ratio 1:4 (125 μL sperm samples and 500 μL PF). For the seek of simplicity we preferred to evaluate many fold peritoneal fluid pools than the single PF, accordingly, a pool of peritoneal fluids was prepared from either groups by mixing equal volumes of fluid samples taken from patients with endometriosis.

Sperm viability [Eosin-Nigrosin staining technique (Björndahl et al., 2003)], total and progressive motility variables were evaluated at 0, 3 (Weidinger et al., 2003) and 24 h (Mansour et al., 2009) by manual counting of sperm observed under phase contrast microscopy.

After 3 h no significant difference in viability was observed in comparison with starting conditions (after swim up, T0), while after 24 h viability was significantly decreased with respect to T0, in the presence of HTFM, of peritoneal fluid from both women with endometriosis and control patients (*p* < 0.05), but no difference was observed when comparing viability of spermatozoa incubated for 3 h or 24 h in either PF-C or PF-EMS, to that of sperm incubated for the same period in HTF medium alone (Figure 3).

### LAD2 Human Mast Cell Line

The human MC line LAD2 was kindly provided by Prof. Carlo Pucillo (Department of Medicine Medical Area, University of Udine, Udine, Italy). The cell line was established from bone marrow aspirates of a patient with MC sarcoma leukemia and is closely related to hMC (Kirshenbaum et al., 2003). This cell line has been widely used as a suitable model for analyzing mast cell functions particularly degranulation and cytokine secretion (Gage et al., 2009; Chan et al., 2014; Ramis et al., 2015) suggesting that they are endowed with a well-defined secretory apparatus and can be used to investigate the mechanism of activation of their secretory pathways. Furthermore LAD2 have increased expression of tryptase, which represent the mast cell mediator of interest in our study, with respect to other human mast cell line HMC-1 (Kanerva et al., 2009; Guhl et al., 2010; Rådinger et al., 2010).

LAD2 cells were grown in serum-free medium StemPro-34 (Gibco, Grand Island, NY) supplemented with 2 mM L-glutamine (Gibco), 1% penicillin-streptomycin (Gibco), and 100 ng/mL human stem cell factor (PeproTech Inc., Rocky Hill, NJ, United States) in a humidified atmosphere of 5% CO2 in air at 37°C. LAD2 were periodically tested for c-Kit expression on the cell surface by flow cytometry (FACScan, Becton Dickinson, San Diego, CA, United States). LAD2 cells were suspended at 1–2 × 10^6^/mL in Tyrode’s buffer containing 0.02% BSA (TyB).

### *In vitro* Experimental Model of MC-Sperm Interaction in the Peritoneal Environment

LAD2 cells were incubated (30 min at 37°C) with or without highly motile sperms (1:3–5 ratio of cells to sperms), in Tyrode’s buffer containing 0.02% BSA (TyB) or in the presence of 10% pool PF-EMS or PF-C, then spun down at 250 × *g* for 7 min. With the aim of evaluating if sperm represents a secretory stimulus for MC, supernatants (SN) and pellets obtained after centrifugation were evaluated for tryptase and β-hexo activity (degranulation assay, see below).

### Degranulation Assay

Tryptase and β-hexo activity (degranulation assay) were evaluated in the supernatant (SN) and pellets using as enzyme substrates, tosyl-gly-pro-lys-p-nitroanilide (0.25 mM final concentration) for tryptase (see above) and 4-nitrophenyl N-acetyl-β-D-glucosaminide (0.5 mM final concentration) for β-hexo [as previously described (Gri et al., 2008; Medic et al., 2008)]. The enzymatic activity was measured in an ELISA reader (Bio-tek instruments INC) at 450 nm. The extent of degranulation was calculated as the percentage of free pNA (for tryptase) or 4-p-nitrophenol (for β-hexo) absorbance in the supernatants taking the sum of the activities found in the supernatants and in cell pellets solubilized in hexadecyl trimethyl ammonium bromide (CTAB) (Sigma-Aldrich, United States) 0.05%, as 100%. Of note, we choose mainly β-hexo activity as a marker of LAD2 degranulation since tryptase activity in PF was too high and covered up the secretory response of LAD2. 4-p-nitrophenol absorbance values were adjusted subtracting the β-hexo activity of sperms, PF-EMS and PF-C alone. A positive control was obtained by cell stimulation with compound 48/80 (10 μg/mL final concentration), a well-known cationic mast cell stimulating agent, for 30–60 min.

### SEM Analysis of MC-Sperm Interaction

At the end of the incubation carried out for the degranulation assay, two hundred microliters of the cell suspension (see paragraph *In vitro experimental model of MC-sperm interaction in the peritoneal environment*) were diluted with PBS (Dulbecco’s modified phosphate buffered saline (PBS) (Sigma-Aldrich, United States) and placed on a poly-L-lysine-coated glass coverslip (18 mm diameter) (Menzler Glasser). The immobilized cells were allowed to adhere to the coverslip over a 30-min incubation period on ice. The supernatant was decanted, and adherent cells were fixed in 2% glutaraldehyde PBS pH 7.4 at room temperature for 30 min. Fixation was followed by rinsing in PBS and then dehydration through graded ethanols. Samples were transferred to a critical point dryer (Bal-Tec; EM Technology and Application, Furstentum, Liechtenstein) in 100% ethanol and dried through CO2. Coverslips were mounted on aluminum sample stubs and gold coated by sputtering (Edwards S150A apparatus, Edwards High Vacuum, Crawley, West Sussex, United Kingdom).

Cells were observed under both low (X 2,000–4,000) and high (X 35,000–40,000) magnification and representative areas were photographed. SEM micrographs are acquired by a Leica Stereoscan 430i scanning electron microscope (Leica Cambridge Ltd., Cambridge, United Kingdom). In each specimen prepared from two different experiments more than 100 SEM fields, including more than 600 LAD2s, were scored for the presence of adherent sperms. The site of sperm-LAD2 interaction, head/midportion (HM) or tail, was also specified.

### Statistical Analysis

Statistical analysis was performed using Graph Pad Prism (Graph Pad Software Inc., La Jolla, CA, United States). The data (levels of blood parameters, PF cellular, and soluble parameters, number of interacting LAD2 and number of LAD2-interacting sperms and the percentage of degranulation) are presented as mean ± Standard Error (SE) or Standard Deviation (SD) (accordingly to the number of the sample scored). The Kolmogorov–Smirnov test was applied to assess the normality of the studied data. Independent samples Student *t*-test (unpaired) was used to compare data between the two groups. Kruskal–Wallis test was used to evaluate differences in spermatozoa parameters and, when statistically significant differences were present, Dunn’s *post hoc* was used to individuate group differences. In all instances, the level of significance for statistical analysis was set at 5% (*p* < 0.05). Power analysis was made using G^∗^Power software (Faul et al., 2007).

Power analysis was performed to assess feasibility of the study: considering effect size = 1.15 and *a* = 0.05, power is equal to 0.79, indicating that highly probable to find statistical significant differences.

## Results

### Blood Cell Counts and Peritoneal Fluid Characterization of the Cellular Components

Table 1 shows white blood counts performed before surgery. The analysis of blood counts showed no statistically significant difference in the number of total circulating leukocytes and different leukocyte subpopulations between the two groups, except that for basophilic granulocytes, whose relative percent (compared to total leukocytes) was significantly lower (*p* = 0.021) in the study group (mean ± SD: 0.54 ± 0.23) as compared to the control group (mean ± SD: 0.87 ± 0.35).

**TABLE 1:** Demographic and clinical characteristics of patients in the study group: infertile women with endometriosis (EMS, *N* = 11) and control group: fertile women without EMS (C, *N* = 9).

		**Blood (CBC, complete blood count)**					
		**Leukocytes**					
	**Age (years)**	**(10^6^/mL)**	**Neut %**	**Lymph %**	**Mono %**	**Eos %**	**Bas %**
Control (C) group (*n* = 9)	38.4 ± 6.1	7.9 ± 2.3	58.1 ± 8.9	30.3 ± 6.3	4,8 ± 0.9	3.9 ± 2.5	0.87 ± 0.35
Study (EMS) group (*n* = 11)	32.3 ± 4.9	6.6 ± 0.9	54.0 ± 6.6	34.2 ± 5.9	6.5 ± 2.4	3.0 ± 1.3	0.54 ± 0.23
Statistical analysis	*ns*	*ns*	*ns*	*ns*	*ns*	*ns*	*p* = 0.021

*Neut, neutrophils; Lymph, lymphocytes; Mono, monocytes; Eos, eosinophils and Bas, basophils. Values are the mean ± SD. Statistical analysis: Student *t*-test.*

**FIGURE 1:**
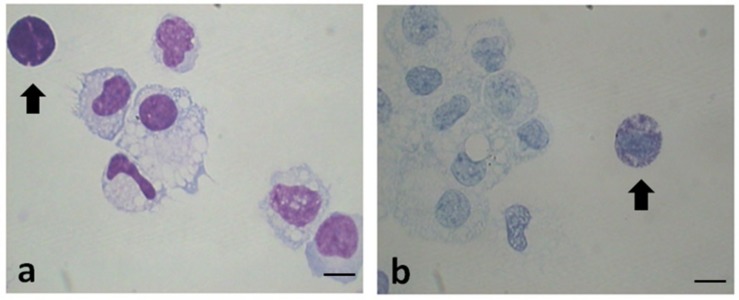
Representative images of cytocentrifuged smears of PF (EMS) stained with Diff-Quick **(a)** and with Toluidin blue (TB) **(b)**. Mast cells/basophils are indicated by the arrows. Original magnification 1,000×. Scale bars = 10 μm.

Table 2 shows the data related to the characteristics of peritoneal fluid of the study group and the control group: volume (mL); cell concentration (expressed in 10^6^cells/mL); total cells (10^6^cells) and percentages for different leukocyte populations evaluated on cytocentrifuged samples of peritoneal fluid stained using the Diff-Quick (DQ) (Figure 1a). To evaluate specifically the population of MC the Toluidin Blue (TB) staining (Figure 1b) was also performed and the results expressed as % of TB positive cells (Table 2). There were no significant differences (*p* = 0.158) neither between the volumes of the PF (mL) in the study group (mean ± SD: 10.1 5.7; *n* = 11) and control groups (mean ± SD: 8.7 ± 3.5; *n* = 9), nor between the total cell count (expressed in 10^6^ cells/mL) of the PF in the study group (1.6 ± 1.3; *n* = 11) and the control group (1.7 ± 1.3; *n* = 9). Furthermore, the PF counts (performed on Diff-Quick stained PF cytocentrifugates) show no statistically significant difference in the percentage of different subpopulations between the two groups with except of neutrophils. As expected (Wang et al., 2018) the percentage of the latter cells resulted significantly increased on the average.

**TABLE 2:** Cytological peritoneal fluid characterization in the study and control group.

			***Diff Quick stain* TB**					
	**Vol PF (mL)**	**10^6^ cells/mL**	**MΦ/mono %**	**Lymph %**	**Neut %**	**Eos %**	**Meso %**	**MC/baso**
Control (C) Group (*n* = 9)	8.7 ± 3.5	1.7 ± 1.3	73.0 ± 10.4	16.0 ± 8.5	8.6 ± 4.5	1.1 ± 0.5	1.3 ± 0.8	0.5 ± 0.5
Study (EMS) Group (*n* = 11)	10.1 ± 5.7	1.6 ± 1.3	56.3 ± 13.4	14.1 ± 10.0	24.6 ± 7.0	2.5 ± 3.8	2.5 ± 5.6	2.8 ± 3.2
Statistical Analysis	*ns*	*ns*	*ns*	*ns*	*p* < *0.05*	*ns*	*ns*	*ns*

*Neut, neutrophils; Lymph, lymphocytes; MΦ/Mono, macrophages/monocytes; Eos, eosinophils; Bas, basophils; MC, mast cells and Meso, mesothelial like cells; TB, toluidine blue; n, patient number. Values, reported as percentage of total cell counted (always more than 200), are the mean ± SD. Statistical analysis: Student *t*-test.*

**FIGURE 2:**
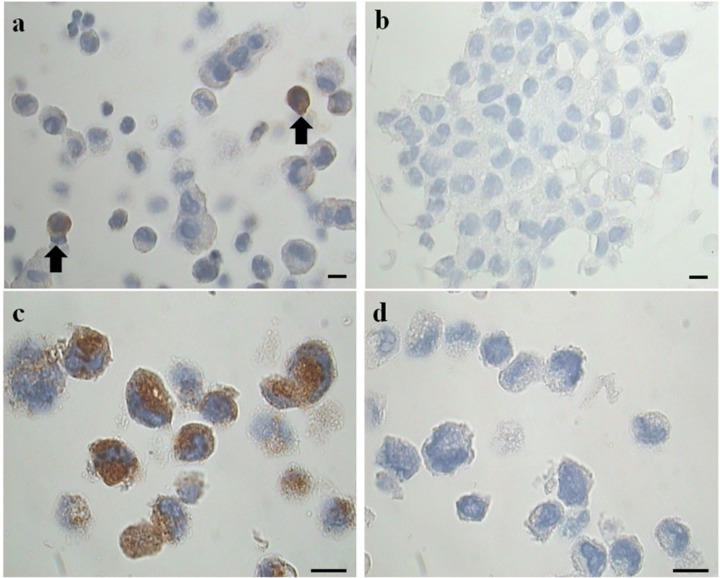
Representative images of immunohistochemical analysis for tryptase on cell blocks’ sections. **(a)** Two tryptase positive cells (from the EMS study group) (arrows). **(b)** Negative control (primary antibody omitted). LAD2 cells in **(c)** represent a positive maker control for tryptase, in **(d)** the negative control (primary antibody omitted) is shown. Original magnification 1,000×. Scale bars = 10 μm.

**FIGURE 3:**
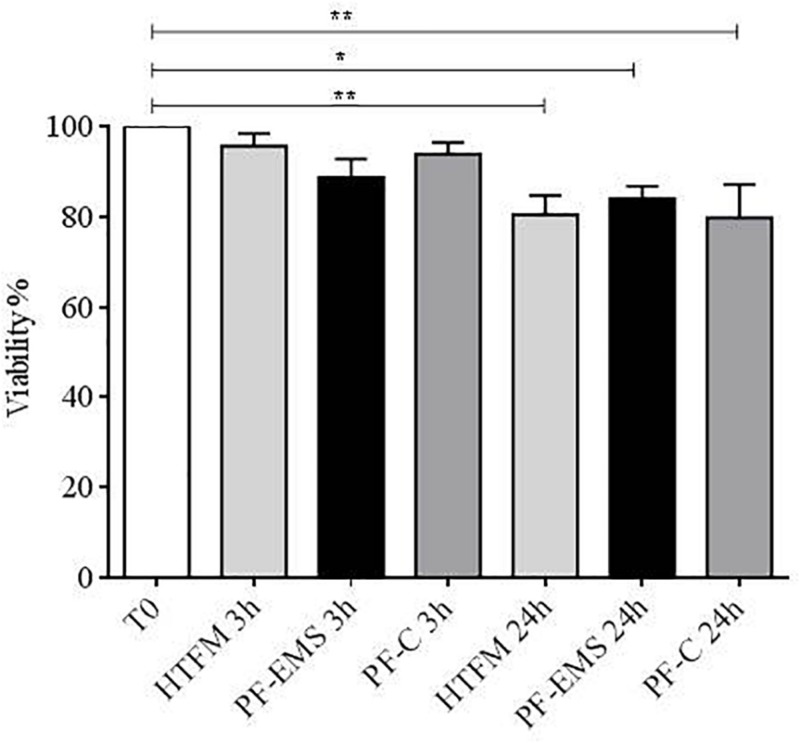
Sperm viability (mean % + SE) after swim-up (T0), after 3 and 24 h incubation in HTF medium (HTFM), peritoneal fluid from women with endometriosis (PF-EMS) and from controls (PF-C). T0 vs. HTFM 3 h: *p* = 0.9271; T0 vs. PF-EMS 3 h: *p* = 0.2077; T0 vs. PF-C 3 h *p* = 0.7543; T0 vs. HFTM 24 *p* = 0.0083; T0 vs. PF-EMS 24 h *p* = 0.0386; T0 vs. PF-C 24 h *p* = 0.0061. ^∗^*p* < 0.05; ^∗∗^*p* < 0.01.

Table 2 also shows the data related to the MC counts evaluated on PF cytocentrifugates stained using TB, which makes recognizing MC more easy (EMS vs. C mean % ± SD: 2.8 ± 3.2 vs. 0.5 ± 0.5 *p* = 0.616). MC population is reduced on the average in the control group, but these values were not significantly different.

To clarify the results obtained with the cytocentrifugates the remaining volume of PF was fixed in formalin (4%) and cell blocks were prepared. Serial sections (3 μm) obtained from these cell-blocks were processed for immunohistochemical analysis for tryptase, a specific MC marker (Figures 2a,b), using LAD2 cells as a positive marker control (Figures 2c,d). The results, expressed as percentage of tryptase positive cells on total cells counted showed that: the study group is characterized by a percentage of tryptase-positive cells (MC) significantly (*p* = 0.044) higher (mean % ± SD: 1.2 ± 0.6; *n* = 11) compared to the control group (mean % ± SD: 0.6 ± 0.3; *n* = 9). These findings agree with the trend revealed by the preliminary analysis of cytocentrifuged samples stained with TB reported in Table 2. Of note the density of tryptase-positive cells in the endometriotic lesions (Supplementary Figure S1 and Supplementary Table S2) is comparabe with those reported in the literature (Konno et al., 2003; Kempuraj et al., 2004; Sugamata et al., 2005; Anaf et al., 2006; Kirchhoff et al., 2012; Paula et al., 2015).

### Peritoneal Fluid Characterization of Soluble Mediators

There were no significant differences (*p* = 0.572) between the protein concentration (as measured by the Bradford method) of the PF between cases (mean ± SD: 1.19 ± 0.04 mg/mL) and controls (mean ± SD: 1.21 ± 0.06 mg/mL) (Table 3).

**TABLE 3:** Biochemical characterization of the peritoneal fluid in the study and control group.

	**Control (C)**	**Study (EMS)**	**Statistical**
	**Group (*n* = 9)**	**Group (*n* = 11)**	**analysis**
Protein concentration (mg/ml) *Mean* ± *SD*	1.21 ± 0.04	1.19 ± 0.06	*ns*
β-hexosaminidase enzyme activity (AU/ml) *Mean* ± *SD*	5.9 ± 1.3	6.5 ± 1.3	*ns*
Tryptase enzyme activity (AU/ml) *Mean* ± *SD*	3.0 ± 0.8	4.2 ± 1.2	*p* = 0.031
Tryptase concentration (ng/ml) *Mean* ± *SD*	12.3 ± 2.3	16.4 ± 3.9	*p* = 0.023

*Arbitrary units (AU) are expressed as: absorbance of 4-nitrophenol/ml for β-hexosaminidase; absorbance of *p*-nitroaniline/ml, for Tryptase. For more details see text. Statistical analysis: Student *t*-test, two tailed unpaired.*

The PF study group (EMS) is characterized by a statistically significant (*p* = 0.031) increased tryptase enzymatic activity (mean ± SD: 4.2 ± 1.2) compared to the control group (mean ± SD: 3.0 ± 0.8) (Table 3). The concentration of tryptase expressed as ng/mL of PF is also shown in Table 3. Similarly to what was found for the enzymatic activity of the tryptase in the PF, the study group was characterized by a concentration of tryptase (mean ± SD: 16.4 ± 3.9) significantly higher (*p* = 0.023) than that of the control group (mean ± SD: 12.3 ± 2.3).

Results reported in Table 3 show that there were no statistically significant differences between the peritoneal levels of β-hexosaminidase enzymatic activity in the two groups (*p* = 0.408). This result is not surprising, since this enzyme is not MC-specific as tryptase is.

### Modulation of Human Sperm Function by Peritoneal Fluid

Motility-enriched sperm samples (*n* = 19) were incubated in HTFM containing 0.05% BSA or peritoneal fluid pools, respectively, obtained by mixing equal volumes of fluid samples taken from patients with endometriosis [PF-EMS (#11): final tryptase concentration = 14.7 ng/mL] and those collected from women endometriosis-free [PF-C (# 9): final tryptase concentration = 11.1 ng/mL].

**FIGURE 4:**
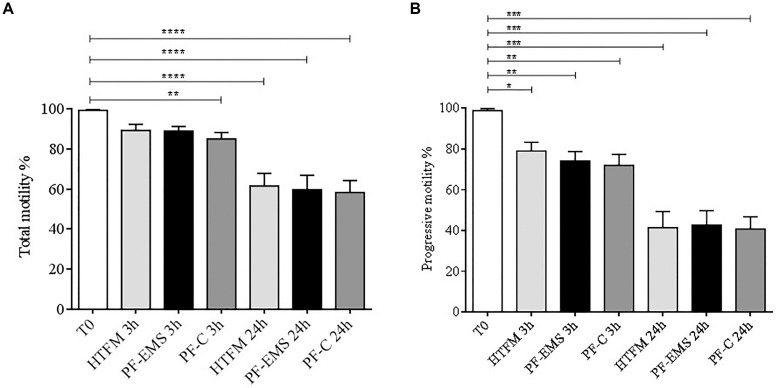
Total **(A)** and progressive **(B)** sperm motility (*mean %* ± *SE*) after swim-up (T0 starting condition), after 3 and 24 h incubation in HTF medium alone (HTFM), peritoneal fluid from women with endometriosis (PF-EMS) and from controls (PF-C). In (A) T0 vs. HTFM 3 h: *p* = 0.4617; T0 vs. PF-EMS 3 h: *p* = 0.0642; T0 vs. PF-C 3 h *p* = 0.0058; T0 vs. HFTM 24 h *p* < 0.0001; T0 vs. PF-EMS 24 h *p* < 0.0001; T0 vs. PF-C 24 h < 0.0001. In **(B)** T0 vs. HTFM 3 h: *p* = 0.0133; T0 vs. PF-EMS 3 h: *p* = 0.0043; T0 vs. PF-C 3 h *p* = 0.0027; T0 vs. HFTM 24 *p* < 0.0001; T0 vs. PF-EMS 24 h *p* < 0.0001; T0 vs. PF-C 24 h *p* < 0.0001. In both figures ^∗^*p* < 0.05; ^∗∗^*p* < 0.01; ^∗∗∗^*p* < 0.001 and ^****^p < 0.0001.

Total and progressive sperm motility (Figures 4A,B) was significantly decreased in comparison with starting conditions (T0) both after 3 and 24 h incubation period in all condition tested (*p* < 0.05). Anyway, no significant differences were observed when comparing total and progressive motility of spermatozoa incubated for 3 or 24 h in either PF-C or PF-EMS, to that of sperm incubated for the same period in HTF medium alone.

### SEM Analysis of LAD2-Sperm Interaction

**FIGURE 5:**
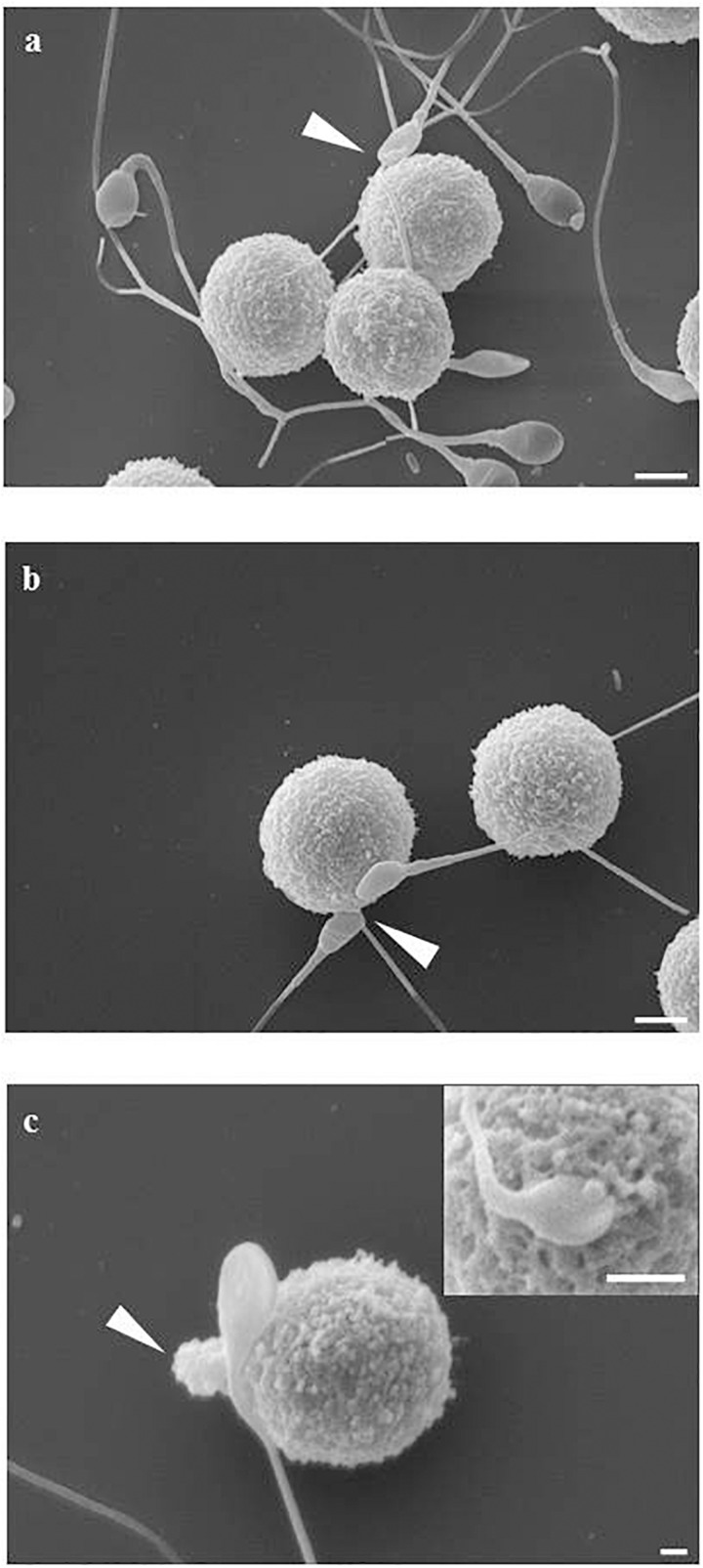
LAD2-sperm interaction evaluated by SEM analysis. **(a)** represents the LAD2-sperm interaction in the presence of PF-C. At least four tail-LAD interactions are apparent with three LAD, while only one head-LAD interaction can be seen (arrow). **(b)** is representative of LAD2-sperm interaction in the presence of PF-EMS, two sperms in contact with one LAD2 are visible (arrow). **(c)** and inset shows two intimate HM interactions between sperm and LAD surface in the presence of PF-EMS. In both cases, extracellular secretion can be seen, as either massive extrusion [arrow in **(c)**] or single granule secretion [inset in **(c)**]. Magnification bars: in **(a,b)** = 3 μm, in **(c)** = 1 μm and in the inset of **(c)** = 3 μm.

The mast cell line LAD2 was incubated for 1 h at 37°C alone or with sperms (cells ratio: sperms = 1:3–5) in TyB alone or supplemented with either 10% of peritoneal fluid of study (PF-EMS) or control (PF-C) group. LAD2 cells-sperm interaction was evaluated by scanning electron microscopy (SEM) in samples prepared from two separate experiments. When incubated for 1 h LAD2 cells and sperms frequently show cell to cell adhesive interaction. This interaction is realized either through the sperm head/midportion (HM) or the sperm tail as previously described for macrophages (Blanco et al., 1992). Figure 5 summarizes these types of interaction as monitored by SEM analysis. Figure 5a represents the LAD2-sperm interaction in the presence of PF-C. At least four tail-LAD2 interactions are apparent with three LAD2, while only one head-LAD2 interaction can be seen (arrow). Conversely, in Figure 5b, which is representative of LAD2-sperm interaction in the presence of PF-EMS, two sperms in contact with one LAD2 are visible (arrowhead).

With the purpose of evaluating in detail the extent of LAD2-sperm interaction we analyzed more than 100 SEM fields and scored the number of sperm-head/midportion (HM) and sperm-tail interaction with LAD2. Table 4 shows the result of this analysis. On average, the total sperm-interacting LAD2 (either by HM or by tail) in the presence of PF-EMS accounted for 2.3 cells/field while the interacting cells in the presence of PF-C were 1.3 (ns). The HM sperm-LAD2 interaction in PF-EMS were significantly higher, accounting for 1.2 interactions/field with respect to that observed in the presence of PF-C (0.7 interactions/field). The difference was statistically significant with *p* < 0.05. Conversely the tail sperm-LAD interaction was almost the same in either PF-EMS or PF-C and was not further considered. Figure 5c shows two intimate adhesions between sperm and LAD2 surface in the presence of PF-EM. In both cases, extracellular secretion/degranulation can be seen, as either massive extrusion (arrow in c) or single granule secretion (inset in c).

**TABLE 4:** Quantitative and qualitative analysis of LAD2-sperm interaction.

	**Field**	**LAD2**	**LAD2/Field**	**InteractingLAD2/**	**HM interactions/**	**Tail interactions/**
	**scored (n)**	**scored (n)**	**(*mean* ± *SE*)**	**field (*mean* ± *SE*)**	**field *(mean* ± *SE*)**	**field (*mean* ± *SE*)**
PF-C	100	719	8.8 ± 0.50	1.3 ± 0.3	0.7 ± 0.2	1.2 ± 0.3
PF-EMS	120	646	7.3 ± 0.5	2.3 ± 0.3	1.2 ± 0.1	1.3 ± 0.2
Statistical analysis				*ns*	*p* < *0.05*	*ns*

*HM, head/midportion. Values are the mean ± SE, obtained from the evaluation of two different experiments. Statistical Analysis: Student *t*-test, two tails unpaired.*

Since degranulation is a reliable marker of MC activation, we assayed cell culture supernatants for the presence of β-hexo (β-hexo), an enzyme located in MC granules.

### LAD2 Degranulation Induced by Sperm Interaction

β-hexo is used as a typical marker of mast cell degranulation *in vitro*. Table 5 shows that the amounts of β -hexo secreted by LAD2 cells is significantly higher after the addition of motile sperm to resting cells (mean % ± SD: 6.3 ± 0.7 *n* = 3 vs. 4.2 ± 0.9 *n* = 5 *p* = 0.0119) (Table 5).

**TABLE 5:** Secretory response of LAD2 induced by sperm interaction.

	**Degranulation:**		
	**% β-hexo release**		
	**(*mean* ± *SD*)**	***n***	**Statistical analysis**
Resting LAD2	4.2 ± 0.9	5	
LAD2+sperm	6.3 ± 0.7	3	[1] vs. [2]: *p* = 0,0119
LAD2+sperm in PF-C	7.0 ± 2.0	3	[2] vs. [3]: *ns* [4] vs. [3]: *p* = 0,0007
LAD2+sperm in PF-EMS	16.7 ± 2.5	3	[2] vs. [4]: *p* < 0.0001
LAD2+48/80	17.4 ± 2.1	3	[1] vs. [5]: *p* < 0.0001

*Percent of release of β-hexo. form LAD2 cells alone (Resting LAD2) and challenged (30 min) with sperm in the absence (in TyB) and the presence of pools of PF from EMS (PF-EMS) and control subjects (PF-C). The positive control was obtained by cell stimulation with compound 48/80. β-hexo, β-hexosaminidase. Values are expressed as percentage of enzymatical activity released, taken the total activity of LAD2- βhexo as 100%. For more details see text. Statistical analysis: Dunn’s *post hoc* was used to individuate group difference.*

LAD2 can release also tryptase in the extracellular medium and, likewise to β-hexo, the amount of tryptase secreted by LAD2 cells is higher after the addition of motile sperms to resting cells (% tryptase release, mean of two experiments: 26.3 vs. 15.2, *n* = 2). Anyway we choose β -hexo activity as LAD2-degranulation marker in the following experiments, since tryptase activity in PF was too high and could mask and underestimate the secretory response of LAD2. LAD2 co-incubated (30 min) with human motile sperms in the presence of PF (10%) pool from controls or PF pool from EMS patients were evaluated for β -hexo release (Table 5). While the extent of secretion didn’t change significantly in the presence of PF-C with respect to the presence of sperm only (mean % ± SD: 7.0 ± 2.0 *n* = 3 and 6.3 ± 0.7 *n* = 3 – *p* = 0.5954), it is significantly increased in the presence of PF-EMS (mean % ± SD: 16.67 ± 2.50 *n* = 3 vs. 6.30 ± 0.66 *n* = 3– *p* < 0.0001) and, interestingly, resulted comparable to the secretion induced by 48/80, a potent mast cell-secretagogue, reported for comparison as a positive control (Table 5). However, considering that the ratio between the percent of LAD2 degranulation (% degranulation) and the percent of LAD2 interacting with head-MD of sperms (% HM interacting LAD2) (Supplementary Table S1) is almost the same either in the presence of PF-EMS or PF-CTRL), it appears that the higher sperm-induced LAD2 degranulation in PF- is almost completely dependent on the HM event number.

## Discussion

Endometriosis (EMS) is a common disease among women of reproductive age and is frequently associated with infertility (Gupta et al., 2008). However, the pathogenesis of this disease is still unknown, so that in several cases the available treatments for symptoms are ineffective (Zito et al., 2014). Increasing evidence supports an involvement of MC either in the inflammatory process of EMS (Kirchhoff et al., 2012) and infertility (Meineke et al., 2000; Weidinger et al., 2003, 2005; Roaiah et al., 2007; Haidl et al., 2011; Menzies et al., 2011). MC are normal constituents of the human myometrium and endometrium and are present in the female genital tract (Sivridis et al., 2001; Hunt and Lynn, 2002). In particular, MC was demonstrated in the oviduct wall (Hunt and Lynn, 2002) and under the lining epithelium of human Fallopian tubes (Weidinger et al., 2003). Endometriotic lesions are characterized by high numbers of degranulated MC (Konno et al., 2003; Kempuraj et al., 2004; Sugamata et al., 2005; Anaf et al., 2006; Kirchhoff et al., 2012; Paula et al., 2015). Mast cells leave evidence, a “fingerprint,” of their participation in acute clinical events, that is an elevation in levels of their secreted mediators/metabolites. Of these, tryptase is currently one of the diagnostic criteria for mast cell activation (Butterfield et al., 2018). While clearly showing MC in the female genital tract, it remains unclear whether or not tryptase from the tubal or uterine MC can reach the lumina of these organs. Up to now the concentration of tryptase in the uterus or Fallopian tubal fluid has not been reported. The presence of tryptase in the female genital tract have been only supposed from its presence in the follicular fluid. In this study, we characterized the peritoneal environment associated with endometriosis and infertility (comparing it to control, fertile and endometriosis-free conditions), as regards the MC cellular component and its main product: tryptase.

Our data quantify, for the first time, the presence of an increased number of tryptase-positive cells/MC in the peritoneal fluid of infertile EMS affected women. We also found a significantly reduced number of blood basophils in EMS-group. These cells share with MC the hematopoietic precursor (Yu et al., 2018) and many other features (Marone et al., 2005) and may in principle be involved in EMS pathogenesis, as well. We could speculate a mobilization of basophils from the circulation to the peritoneal cavity. However, this contribution is difficult to evaluate since standard staining techniques are not able to distinguish MC from basophilic granulocytes. Anyway, since normal blood basophils express only trace amounts of tryptase (Li et al., 1998; Samorapoompichit et al., 2001), which has been the main tool and target of our research, and the main diagnostic criteria for identifying mast cell (Butterfield et al., 2018), their contribution was not further considered and should deserves a dedicated research. The increase of MC in the peritoneal environment we found does not prove by itself that MC are involved in the EMS pathogenesis, but is in agreement with the increment of tissue MC population in EMS condition described by other authors (Konno et al., 2003; Kempuraj et al., 2004; Sugamata et al., 2005; Anaf et al., 2006; Kirchhoff et al., 2012; Paula et al., 2015).

Accordingly, the peritoneal fluid in infertile-EMS conditions was characterized by higher levels of enzymatically active tryptase, the main MC product. Higher EMS peritoneal fluid levels of tryptase could be ascribed not only to the increment of peritoneal MC population but also to that of tissue population. These cells are responsive to many different receptors and can be activated by various kind of stimuli (Redegeld et al., 2018). Now, the physical interaction with sperms should be added to the list. The released mediators could reach in the peritoneum a high level which in principle could affect sperm motility and fertility as previously suggested. Human recombinant tryptase has been shown to inhibit sperm motility *in vitro* (Weidinger et al., 2003, 2005) and accordingly tryptase has been supposed as a yet unrecognized factor capable of influencing sperm fertilizing ability. Anyway, up to now tryptase levels have been evaluated only in human seminal plasma of andrological patients (4.18 ± 1.95 ng/mL) and in human follicular fluid (ranging from 1.60 to 3.73 ng/mL), where they results far below the lower level capable of inhibit sperm motility *in vitro* (10 ng/mL). Accordingly, seminal fluid’s levels of tryptase were not correlated to sperm motility (Weidinger et al., 2003).

Infertility is a condition associated with EMS and the effect of peritoneal fluid associated with the condition of endometriosis on sperm motility has been a long controversial topic: some experimental evidence support a decrease in sperm motility in this environment (Aeby et al., 1996; Oral et al., 1996; Liu et al., 2000; Luo et al., 2008; Xu et al., 2008), while other authors report no effect (Chen et al., 1997; Sharma et al., 1999; Munuce et al., 2003).

Interestingly, the concentration of enzimatically active tryptase that we found in the peritoneal fluid of both study (EMS: 16.4 ± 3.9 ng/mL) and control group (C: 12.3 ± 2.3 ng/mL) was higher than those reported in human seminal plasma of andrological patients and human follicular fluid, and higher than the minimum concentration capable of inhibiting *in vitro* sperm motility (Weidinger et al., 2003). In spite of this we didn’t find any differences when analyzing sperm viability and motility (total and progressive) in the presence of tryptase enriched peritoneal fluid associated with endometriosis and infertility with respect to control, fertile and endometriosis-free conditions (nor to control medium alone), both in short and long term incubation. Our data are in agreement with previous reports showing the absence of any effect of EMS peritoneal fluid on sperm motility (Chen et al., 1997; Sharma et al., 1999; Munuce et al., 2003) and suggest that tryptase levels, able of inhibiting sperm motility *in vitro*, are ineffective in the peritoneal environment. Anyway we cannot exclude that tryptase (or other still unrecognized MC mediators) could modulate other sperm function *in vivo*, eg. acrosome reaction (Arumugam, 1994; Munuce et al., 2003) or sperm-oocyte interaction (Aeby et al., 1996; Wong et al., 2001; Caille et al., 2012). While the present report was focused on sperm motility in the presence of EMS-PF, the possible effects of tryptase on sperm functions, will be the subject of future research.

Furthermore, Cincik and Sezen previously reported that the presence of MC in semen, by itself negatively affects sperm motility (Cincik and Sezen, 2003). Accordingly, since the number of tryptase-positive cells/MC increases in the peritoneal fluid of infertile EMS affected women we decided to investigate the possible interaction between these cells and sperm in the peritoneal environment. We co-incubated mast cells (cell line LAD2) and sperms in a pool of PF (10% v/v) obtained from infertile women with EMS or from fertile endometriosis-free controls. Sperm was shown to interact more with LAD2 in the presence of PF-EMS. Furthermore LAD2-sperm interaction was shown to induce a secretory response from LAD2 cells (by morphological and secretory evaluations), which was significantly higher in EMS conditions. We suggest that this secretory response could contribute to increase the level of tryptase present in PF-EMS.

## Conclusion

In conclusion, on the basis of our findings it appears unlikely that tryptase enriched peritoneal fluid could affect sperm motility.

Anyway the present study presents some reasons for caution, as follows:

– The sample size was limited. A broader study concerning different stages of endometriosis would increase the value of our results.
– Human resident peritoneal mast cells are not readily purified. To overcome this limitation in studying in human beings the MC-sperm interaction, we used the most differentiated human mast cells line available, endowed with a high content of tryptase.
– The sperm parameters analyzed were limited to viability and motility, so we cannot exclude that tryptase (or other still unrecognized MC mediators) could modulate other sperm functions *in vivo*, contributing to the infertility associated with endometriosis.

The novelty reported in this paper is that the presence of a tryptase rich PF can stimulate the sperm-mast cell interaction and induce degranulation from these cells. However, even if tryptase-positive cells are present in a higher percentage in the PF-EMS, they remain quantitatively underrepresented, and therefore their strong interaction with the sperm in the EMS conditions couldn’t be enough to affect sperm function. We think that this strong physical and functional mast cell-sperm interaction could be more effective in the male genital tract where MC mediated negative effects on sperm functions has been reported but not clarified so far and require further investigation (Agarwal et al., 1987; Hashimoto et al., 1988; Nagai et al., 1992; Hussein et al., 2005; El-Karaksy et al., 2007; Haidl et al., 2011; Menzies et al., 2011; Windschüttl et al., 2014) One potential benefit to assisted reproductive clinics could derive from targeting mast cells - sperm interaction to treat male infertility due to testicular pathologies associated with inflammation and germ cell loss, as recently suggested (Mayerhofer et al., 2018).

## Data Availability Statement

The datasets generated for this study are available on request to the corresponding author.

## Ethics Statement

The studies involving human participants were reviewed and approved by the Comitato Indipendente di Bioetica (CIB)-Burlo Garofolo. The patients/participants provided their written informed consent to participate in this study.

## Author Contributions

VB developed the idea for the manuscript, formulated the study design, and performed the peritoneal fluid analyses, interpretation of the data, and manuscript drafting. FR, FF, and GR performed the surgical procedures. FC contributed to the statistical analyses. GR conducted the clinical evaluations and contributed to the statistical analyses, data interpretation, and manuscript drafting. MM and SL recruited patients and performed the sperm analysis. FZ and CB performed the immunocytochemical/immunohistochemical staining and data interpretation. FV performed the scanning electron microscopy analysis. ET performed the evaluation with LAD2 cell line. GZ participated in a critical revision and manuscript drafting. All authors have approved the final version of the manuscript.

## Conflict of Interest

The authors declare that the research was conducted in the absence of any commercial or financial relationships that could be construed as a potential conflict of interest.
