# Supplementary figures and images for: Mast Cells in Peritoneal Fluid From Women With Endometriosis and Their Possible Role in Modulating Sperm Function
Source: Front Physiol. 2020 Jan 9;10:1543. doi: 10.3389/fphys.2019.01543 (PMC6964357; doi:10.3389/fphys.2019.01543)

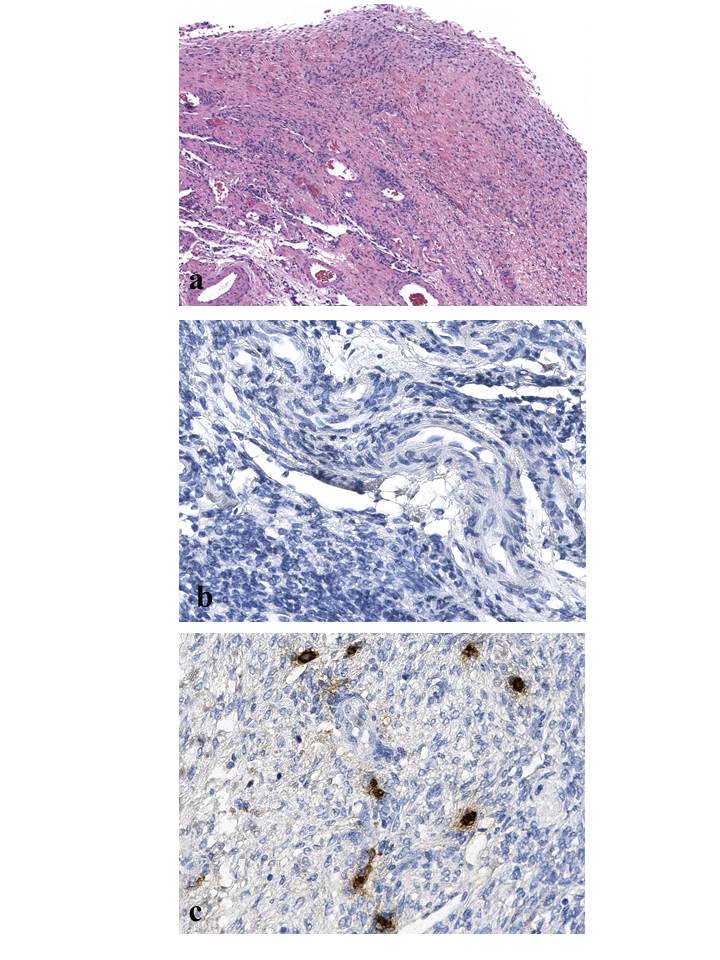

Supplement: Supplementary file 1 [file Image_1.JPEG]
