# Supplementary material for: Mast Cells in Peritoneal Fluid From Women With Endometriosis and Their Possible Role in Modulating Sperm Function
Source: Front Physiol. 2020 Jan 9;10:1543. doi: 10.3389/fphys.2019.01543 (PMC6964357; doi:10.3389/fphys.2019.01543)
Supplement: Supplementary file 2 [file Table_1.docx]

**SUPPLEMENTAL DATA: tables and figure legends**

**S.1 Calculation of the ratio secretion/interaction**

|  | **Degranulation:**  **%βhexo release**  **(mean +SD)** | **%HM interactions on LAD2/field*** | **Ratio:**  % degranulation**____**  % HM interactions |
| --- | --- | --- | --- |
| **Resting LAD2** | 4.2 + 0.9 |  |  |
| **LAD2+sperm** | 6.3 + 0.7 |  |  |
| **LAD2+sperm in PF-C** | 7.0 + 2.0 | **7.6**  **[1.2/7.3]**** | **1.1** |
| **LAD2+sperm in PF-EMS** | 16.7 + 2.5 | **16.4 [0.67/8.8)**** | **1.0** |

***Table S1.*** *Secretory response of LAD2 induced by sperm interaction: percent of release of β-hexo. form LAD2 cells co-incubated (30min) with sperm in the presence of pools of PF from EMS (PF-EMS) and control subjects (PF-C). *The percentage of head/midportion (HM) interactions was expressed as the mean number of HM on LAD2 cells/field taken as 100 %. The ratio degranulation/sperm interaction was calculated as the ratio between the % of degranulation and the % of HM sperm interacting with LAD2. ** values from Table 4.*

**S.2 Immunohistochemical characterization of mast cells in different anatomic locations of endometriosis in patients of the study group.**

Seven EMS patients presenting five different anatomic locations of endometriosis were studied: peritoneum (parietal peritoneum in the ovarian fossa), n=1; ovary, n=3; Fallopian tubes, n=1; rectovaginal septum, n=1; uterosacral ligament, n=1 (Table S2). After resection, all the tissues were fixed in 4% formaldehyde for 12 hours and then embedded in paraffin.

In each specimen, 5 serial sections of 4 µm were obtained. The first section was stained with hematoxylin and eosin to confirm the diagnosis of endometriosis, and the other sections were used for immunohistochemistry and controls. Immunohistochemistry was applied on tissue sections with monoclonal antibodies against tryptase: Anti-human Mast Cell Tryptase monoclonal antibody 15-MOB347 (1:300) (Bio Optica). Detection system: Ultravision Quanto Large Volume Detection System HRP Polymer with Dewax e HIER Buffer H, pH 8 (Bio Optica). Positive reactions resulted in brown-colored staining at the site of target antigen tryptase (Figure S1). Negative controls incubated ommiting the primary antibody did not show any positive staining for tryptase. Positive controls consisted of tonsils. Each tissue slide was scanned with a digital D-Sight Fluo 2.0 microscope and analysed with VISIA Imaging S.r.l. Software.


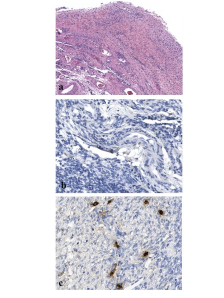


***Figure S1.*** *Photomicrographs of endometriosis biopsies (ovary) immunostained for mast cell tryptase. The first section was stained with hematoxylin and eosin to confirm the diagnosis of endometriosis (panel a), the other sections were incubated with mouse anti-human tryptase monoclonal antibody (15-MOB347 anti. Mast Cell Tryptase, Bio Optica, 1:300). Positive reactions resulted in brown-coloured staining at the site of target antigen tryptase (panel b). Negative controls incubated omitting the primary antibody, did not show any positive staining for tryptase (panel b). Image acquisition: Leitz Laborlux 12 / D-sight system. Original magnification 40x in panel a, 400 x in panels b and c.*

Immunohistochemistry results were expressed as the mean number of mast cells/mm^2^ + SD (Table S2) and compared with those obtained from literature (Kempuraj *et al.* 2004 and Anaf *et al.* 2006).

|  | | |  | |  |  |  |
| --- | --- | --- | --- | --- | --- | --- | --- |
| EMS Patients (n=7) | | **Biopsy site** | **Total mast cells/mm^2^**  *Mean+SD* | | **Tryptase positive cells/mm^2^**  **in literature**  *(Kempuraj et al. 2004^◊^ and Anaf et al. 2006^◊◊^).* | | |
|  | |  |  | | **EMS** | | **Controls**  **(**normal tissues from patients without endometriosis) |
| #EMS 1 | | **Ovary** | **18.2+8.2** | | **7.5+3.0^◊◊^** | | **0^◊◊^** |
| #EMS 2 | |  | **9.6+3.6** | |  |  |  |
| #EMS 3 | |  | **14.9+4.9** | |  |  |  |
| #EMS 4 | | **Fallopian tubes** | **10.1+5.0** | | **nd** | | **nd** |
| #EMS 5 | | **Parietal peritoneum**  **in the ovarian fossa** | **20.9+11.1** | | **3.3+1.1^◊◊^**  **64.0+3.0^◊^** | | **1.5+0.4^◊◊^**  **0^◊^** |
| #EMS 6 | | **Rectovaginal**  **septum** | **10.4+4.9** | | **8.1+2.0^◊◊^** | | **2.5+1,3^◊◊^** |
| #EMS 7 | | **Uterosacral**  **ligament** | **11.6+5.9** | | **10.4+3.0^◊◊^**  **79,6+3^◊^ ; 119,0+6^◊^** | | **0.5+0.2^◊◊^**  **0^◊^** |
| Positive control | | Tonsils | **38.5+16.1** | | - | | - |

***Table S2.*** *Total mast cell numbers in endometriosis lesions. Mast cells were counted in sections from each patient using high power field (mast cells/mm^2^). We obtained density levels of tryptase positive cells (MC/mm^2^) in the endometriotic lesions comparable and even higher of those described by other authors (Kempuraj et al. 2004^◊^ and Anaf et al. 2006^◊◊^).*
